# Quantifying Components of Soil Respiration and Their Response to Abiotic Factors in Two Typical Subtropical Forest Stands, Southwest China

**DOI:** 10.1371/journal.pone.0117490

**Published:** 2015-02-13

**Authors:** Lei Yu, Yujie Wang, Yunqi Wang, Suqi Sun, Liziyuan Liu

**Affiliations:** Key Laboratory of Soil and Water Conservation and Desertification Combating, Ministry of Education, Beijing Forestry University, Beijing, China; Tennessee State University, UNITED STATES

## Abstract

Separating the components of soil respiration and understanding the roles of abiotic factors at a temporal scale among different forest types are critical issues in forest ecosystem carbon cycling. This study quantified the proportions of autotrophic (*R*_A_) and heterotrophic (*R*_H_) in total soil (*R*_T_) respiration using trenching and litter removal. Field studies were conducted in two typical subtropical forest stands (broadleaf and needle leaf mixed forest; bamboo forest) at Jinyun Mountain, near the Three Georges Reservoir in southwest China, during the growing season (Apr.–Sep.) from 2010 to 2012. The effects of air temperature (AT), soil temperature (ST) and soil moisture (SM) at 6cm depth, solar radiation (SR), pH on components of soil respiration were analyzed. Results show that: 1) SR, AT, and ST exhibited a similar temporal trend. The observed abiotic factors showed slight interannual variability for the two forest stands. 2) The contributions of *R*_H_ and *R*_A_ to *R*_T_ for broadleaf and needle leaf mixed forest were 73.25% and 26.75%, respectively, while those for bamboo forest were 89.02% and 10.98%, respectively; soil respiration peaked from June to July. In both stands, CO_2_ released from the decomposition of soil organic matter (SOM), the strongest contributor to *R*_T_, accounted for over 63% of *R*_H_. 3) AT and ST were significantly positively correlated with *R*_T_ and its components (*p*<0.05), and were major factors affecting soil respiration. 4) Components of soil respiration were significantly different between two forest stands (*p*<0.05), indicating that vegetation types played a role in soil respiration and its components.

## Introduction

Soil, the planet’s largest terrestrial carbon pool, sequesters photosynthetically-assimilated carbon through the burial of plant biomass. Most sequestered carbon is eventually returned to the atmosphere by the release of CO_2_ during the process of soil respiration, which represents the second largest flux in the carbon cycle after photosynthesis [1, 2]. Globally, soil respiration releases about 98 billion tons of carbon into the atmosphere every year, which is mainly (90%) from forest soil respiration [3, 4, 5]. Thus, even small changes in soil respiration can cause drastic changes in atmospheric CO_2_ concentration that may potentially influence global climate change [6]. Nevertheless, soil respiration is a complicated biological process driven by many factors. Quantitatively separating forest soil respiration and analyzing the effects of important abiotic factors on soil respiration is a critical and challenging issue, especially in the field of carbon (C) fluxes and cycling in forest ecosystems.

Soil respiration components are usually separated into *R*_A_ (e.g., respiration of plant roots and their symbiotic microorganisms) and *R*_H_ (e.g., respiration of decomposing plant biomass, soil organic matter, litter, and soil animals) [7]. However, the components of soil respiration and the rate at which each component of *R*_T_ contribute to *R*_T_ usually vary in different forest types. Bond-Lamberty and Hanson [8, 9] concluded *R*_A_ in temperate forests as a major component (60%) of *R*_T_, while for subtropical forests the principle component (75%) was *R*_H_ as a fraction of *R*_T_. Therefore, separating components of *R*_T_ contributes significantly to our understanding C storage [10]. Currently, three techniques are commonly used to distinguish the components of *R*_T_ i.e., methods using root exclusion, isotope tracer, and direct measurement. All are designed to use a feature of the soil respiration process to quantitatively define one or more components [11, 12]. However, all three techniques usually involve a compromise related to either low accuracy or inconvenient methods. Root exclusion and direct measurement methods can be done easily, but have limit accuracy since data may contain components of *R*_H_ [13, 14]. While isotope tracer methods are more accurate, the related field techniques are quite difficult to conduct.

Many biological factors (e.g., microbial communities and their activities) [15, 16] or abiotic factors (e.g., substrate supply, litter, and stand structure temperature, moisture, solar radiation, soil pH, nitrogen deposition, and soil texture) [17, 18] influence soil respiration, and interactively affect components of *R*_T_ [19]. As happens with other physiological processes, soil respiration usually responds most strongly to a few dominant factors. Therefore, previous scholars have tried to avoid analyzing the marginal effects of inconsequential factors. Temperature affects almost every aspect of soil respiration—it affects soil respiration components by affecting soil microorganisms, plant roots and the activity of respiratory enzymes [20, 21, 22, 23]. Several types of models, including temperature-respiration models, e.g. linear models, second-order exponential models, exponential models, are usually used to analyze the relationship between temperature and soil respiration [24, 25]; exponentials model are most frequently used [26].

Soil moisture also strongly influences soil respiration. Extremes in soil moisture, high or low, will result in reduced root and/or microbial activity, which consequently inhibit soil respiration [27, 28]. Inversely, when soil moisture approaches field capacity this can enhance soil respiration [29]. Moreover, other abiotic factors, such as soil pH and SR, also influence the soil respiration components to some extent. Sitaula et al. [30] showed that soil pH affects the activities of soil microorganisms, which in turn affects *R*_H_. SR affects soil respiration indirectly by influencing photosynthesis [31]. All of these studies isolated the role of abiotic environmental factors on soil respiration from other factors. However, these factors do not act independently, may interact with each other and mutually affect soil respiration. Therefore, to investigate the effects of abiotic factors on soil respiration, one needs to analyze each factor as well as their interaction, which has seldom been done.

The Jinyun Mountain National Forest Nature Reserve lies within Three-Gorges Reservoir Area along the upper middle Yangtze River. The Reserve has a long history and features the best-preserved typical forest ecosystems in Southwest China. Broadleaf and needle leaf mixed forest and bamboo forest comprise the two main typical forests in this region, covering 77% and 23% of the area, respectively. As the study of the global C cycle accelerates, interest in soil respiration in this region is also increasing. For example, recent research has analyzed the factors affecting soil respiration [32], the sensitivities of soil respiration to temperature [33], and variation features of soil respiration in typical forests [34]. The main objectives are (1) to separate soil respiration components and to understand the reasons they change over time; (2) to identify the contribution of each component to *R*_T_; (3) to clarify the relationships between soil respiration components and abiotic factors; (4) to test the following hypothesis: forest types lead to significant differences in the components of soil respiration.

## Materials and Methods

### 1. Ethics Statement

The study was conducted in the Jinyun Mountain National Nature Reserve in Chongqing, southwestern China. Permission was obtained from the Reserve administration to allow sampling. No rare or endangered wild animals or plants were collected in this experiment. Permit restrictions also required the experiment to be conducted without the use of open flame to prevent forest fires, and without the cutting of tall trees to protect the forest ecosystem from damaged. In addition, the samples were made of common tree species of the subtropical forest system, so sampling had no direct impact on vertebrate survival. This experiment did not employ the use of wild animals or plants as research objects and did not pose a threat to the environment.

### 2. Study site

The study was conducted in Jinyun Mountain National Nature Reserve, located at 29°41′–29°52′N, 106°17′–106°24′E (Chongqing, China), an area with a typical subtropical humid monsoon climate. The elevation ranges from 350 to 951.5 m, the mean annual AT, precipitation and evaporation are 13.6°C, 1611.8 mm, and 777.1 mm, respectively. In addition, the area experiences an average of 104 foggy days annually.

The main vegetation types in this reserve are broadleaf and needle leaf mixed forest (BNMF) and bamboo forest (BF). One experimental site was selected for each vegetation type. The majority of trees in BNMF are *Pinus massoniana*, *Cunninghamia lanceolata*, *Gordonia acuminate*, and *Adinandru bockiana*, the needle leaf forests account for 35.87% and broadleaf forests account for 64.13%, mixed ratio is nearly 4:6. *Phyllostachys pubescens* dominates the bamboo forest. The two sites had no significant difference, and both contained extensive natural yet managed stands (Table 1), which feature flat terrain and deep soil. However, the shallow topsoil is mainly composed of one of two types, acidic yellow soil or sandy soil that supplies substrates for soil respiration (Table 2).

**Table 1:** Basic information of the two forest stands.

Forest types	Site factors			Forest stands						Litter	
	Altitude (m)	Slope aspect	Slope (°)	Origin	Age class	Crown density	TH (m)	DBH (cm)	Density (trees ·ha^-1^)	Thickness (cm)	Dry storage (t·ha^-2^)
**BNMF**	864.5	Northwest	12	Natural	VI	0.90	12.6	11.2	2043	3.0	14.32
**BF**	855.2	Northwest	11	Natural	V	0.85	14.4	10.3	5300	1.4	16.21

BNMF: broadleaf and needle leaf mixed forest; BF: bamboo forest; TH: tree height; DBH: mean tree diameter at breast height.

**Table 2:** The physicochemical properties of the two forest stands.

Forest types	Physical properties (0–15cm)				Chemical properties (0–15cm)			
	Soil bulk density (g·cm^-3^)	Total soil porosity(%)	MWHC (%)	ASWC (%)	Available N (mg·kg^-1^)	Total N (g·kg^-1^)	Organic matter (g·kg^-1^)	pH
**BNMF**	1.03	61	59	40	112.0	2.42	45.4	3.96
**BF**	0.96	64	58	35	97.0	2.55	51.6	4.20

MWHC: maximum water holding capacity; ASWC: average soil water content

### 3. Measurement of total soil respiration

In fall 2009, we randomly defined four 1 m × 1 m sample plots within each observation site for measuring *R*_T_ (*R*_T_ was composed of root, litter and SOM respirations). Each plot was located more than 2 m from any other. A 200 mm diameter and 150 mm tall PVC soil ring was inserted into the soil to a depth of 130 mm in each sample plot before using an instrument to measure soil CO_2_ flux; this simplified the measurement of soil respiration without destroying the soil structure. No treatments or measurements were performed on the soil conditions. Soil respiration rates were measured during growing seasons from April to September, 2010–2012, using an LI-8100 Soil CO_2_ Flux System (LI-COR, Lincoln, NE, USA). Within the observation periods, four days were selected for both the first and the second half of every month, excluding rainy days. On each selected day, measurements were conducted from 6:00 am to 18:00 pm; measurements were carried out at two sites alternatively so that soil respiration was measured at different times for each repetition.

### 4. Measurement of soil respiration components

In this study, root trenching and litter removal treatment were used to separate *R*_A_ and *R*_H_. Usually, *R*_A_ includes respiration of roots and symbiotic microorganisms within plant roots, while *R*_H_ includes respiration of soil microorganisms, SOM and soil fauna [7]. However, in this study root respiration (*R*_r_) was considered as approximately the same as *R*_A_, while respirations of SOM (*R*_SOM_) and litter (*R*_L_) are regarded as approximately the same as *R*_H_. Additionally, the mean soil respiration components were used to reduce systematic errors (SD±1).

At each of two experimental plots, eight 1 m × 1 m sample plots were randomly established; four were randomly selected for root pruning and litter removal, and four had only litter removal. In the former plots, 1.5 m deep trenches (to below the root depth) were dug to prune roots. PVC boards were inserted into the trenches to isolate the root material within the plot. Next, visible living organisms and removable fine roots were then removed from the sample plots; care was taken to keep the soil layers intact. Then, all leaf litter, bulk litter (e.g., fallen branches), and living plants were removed. During the observation periods all new living plants or new litter were removed to avoid affecting respiration measurements. In addition, for five months after root pruning treatment, all sample plots were left otherwise undisturbed to allow the pruned roots within the sample plot to completely decompose prior to soil respiration measurements. Thus, CO_2_ generated during a “pulse” of decomposition was not included in the measurements of *R*_H_ in this study. In the plots with litter removal, only leaf litter and bulk litter was removed from the surface of sample plots; no other treatments were conducted. Soil respiration measurements were carried out using the same method and timeline as for *R*_T_ in both treatments.

### 5. Measurement of abiotic factors

Several abiotic factors were also measured to clarify independent or interactive effects of abiotic factors on soil respiration. ST and SM at 6 cm depth from soil surface were measured using a DDTWS-II Full-Section Reflective Soil Hygrothermograph (Gsome Ltd., Hangzhou, China), while AT and SR were automatically acquired by automated meteorological stations (Dynamax, Houston, TX., USA) in the forest stands. Soil pH at 6 cm deep was measured using a standard method in laboratory (ISO10390:2005). The soil samples of about 1 Kg were collected once during each season for the collection of data related to soil physicochemical properties during the observation period (2010–2012); collections were made at five points for soil samples at 0–15cm depth at each sample site. The soil bulk density, total soil porosity and maximum water holding capacity were measured using the cutting ring method; the average soil water content was measured using the drying method; the available N and total N were measured using the alkaline hydrolysis diffusion method and Semimicro-Kjeldahl method. Organic matter content was measured by the potassium dichromate method.

### 6. Data analysis

Although trenching and litter removal methods were separate the soil respiration components, we could not directly measure all the components. So, the following equations were used to describe *R*_T_ and its components for the two forest stands in this study:

1. Untreated observation plots were used to quantify R_T_, i.e., CO_2_ flux generated by *R*r, *R*_L_, and *R*_SOM_. *R*_T_ can be represented as in Equation (1):

$$R_{\text{T}}\text{=}R_{\text{A}}\text{+}R_{\text{H}}\text{=}R\text{r+}R_{\text{L}}+R_{\text{SOM}}$$ (1)

2. The plots with litter removal treatment were used to quantify the component of soil respiration generated by *R*r and *R*_SOM_, as represented by the CO_2_ fluxes in Equation (2):

$$\text{Soil respiration of litter-free plots }R_{\text{P1}}\text{=}R\text{r+}R_{\text{SOM}}$$ (2)

3. The plots with root pruning and litter removal treatments were used to quantify only the CO_2_ flux from the decomposition of organic matter, as seen in Equation (3):

$$\text{Soil respiration of root and litter-free plots }R_{\text{P2}}\text{=}R_{\text{SOM}}$$ (3)

This study did not directly separate *R*_A_ from roots and respiration of symbiotic bacteria and fungi, so *R*r here was treated as equivalent to *R*_A_. Based on the above equations and this equivalence, we can calculate the relative contributions of *R*_A_ and *R*_H_ to *R*_T_ (Equations 4 and 5):

$$R_{\text{A}}\approx R\text{r }=R_{\text{P1}}-R_{\text{P2}}$$ (4)

$$R_{\text{H}}=R_{\text{T}}-R\text{r}$$ (5)

The contribution of *R*_SOM_ to *R*_H_ was directly determined from *R*_P2_, while the contribution of CO_2_ fluxes generated by decomposition of litter was described in Equation (6):

$$R_{\text{L}}=R_{\text{T}}-R_{\text{P1}}$$ (6)

Correlation analysis was applied to identify relationships between abiotic factors and *R*_T_ or its components in two forest stands. Significance analysis was also used to identify the main abiotic factors influencing *R*_T_ and its components. We used the exponential model (7) to analyze the response of *R*_T_ and its components to temperatures [32, 34]:

$$R_{S}=R_{0}e^{\beta T}$$ (7)

where *R*_S_ represents the soil respiration rate (μmol·m^–2^·s^–1^; any component or total), *T* is temperature (°C), *R*_0_ is the respiratory rate at a reference soil temperature of 0°C, and *β* is the temperature response coefficient.

One-way analysis of variance (ANOVA) was applied to identify significant differences in *R*_T_ or its components between the two forest stands. Multiple comparisons tests were used to identify significant differences between *R*_A_ and *R*_H_, and among *R*r, *R*_L_, and *R*_SOM_. Statistical analyses were carried out using SPSS 20 (SPSS Inc., 1989–2012), and figures were drawn using Origin 8.0 (Origin Lab Inc., Northampton, MA, USA). Soil respiration and environmental factors variable data from the present study are presented in the S1 Dataset. In addition, this study was conducted based on Forestry Standards “Observation Methodology for Long-term Forest Ecosystem Research” of the People’s Republic of China.

## Results

### 1. Dynamic variation of the environmental factors

**Fig 1:**
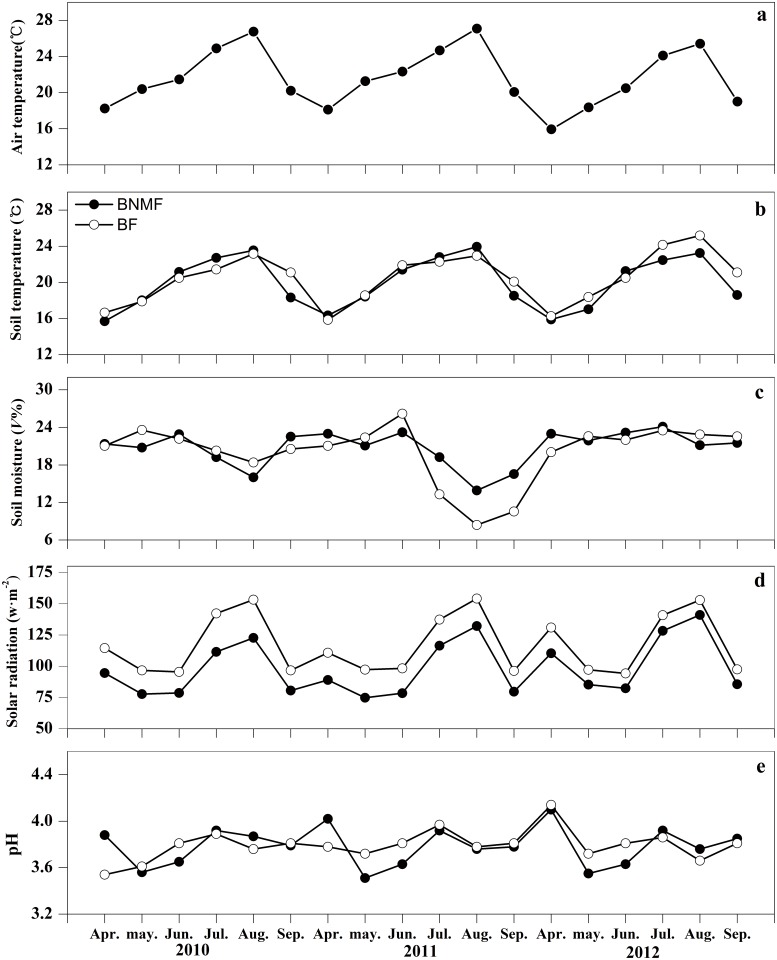
The dynamic variation of monthly average values of abiotic environmental factors of the two forest stands.

During this study, monthly variations of AT, ST and SR exhibited single-peaked curves with peak values generally observed in August. The maximum monthly-averaged AT peaked in August 2011 (27.09 ± 0.54°C), while those of SR also peaked in 2011 for BNMF and BF at 132.1 ± 5.2 w**·**m^–2^ and 154.10 ± 4.5 w**·**m^–2^, respectively (Fig. 1a, 1d). Subject to the control of AT, the monthly average of ST for BNMF and BF also peaked at 23.94 ± 0.48°C in August 2011 and at 25.08 ± 0.62°C in August 2012, respectively (Fig. 1b). Although the average annual SR of the BF was slightly greater than that of BNMF, these data were not statistically significant (*p*>0.05; Table 3). The average annual ST fluctuated within a range of ± 1°C at each site and did not differ significantly between the two forest stands (Table 3). In addition, precipitation caused SM of both forest stands to vary irregularly. However, during this study SM reached a minimum in August in all three years when AT and ST peaked. In August 2011, SM in the BF and BNMF reached its overall minimum for the observation period, at 13.93 ± 0.36 and 8.4 ± 0.43 (*V*%), respectively (Fig. 1c). The average annual SM did not differ significantly between the two forests (Table 3). However, yearly variations were significant: the average yearly SM of BF in 2010 was 19.2% lower than that in 2011 and 2012 (Table 3). Soil pH remained relatively stable, within a range of ± 0.5 units, and it did not differ significantly between two forest stands (Table 3; Fig. 1e).

**Table 3:** The mean values of environmental factors at the two forest stands for grow season (April–September) from 2010 to 2012.

Forest types	Year	Mean year solar radiation(w·m^-2^)	Mean year air temperature(°C)	Mean year soil temperature(°C)	Mean year soil moisture(*V*%)	Mean year pH
**BNMF**	**2010**	94.24±2.8	21.99±0.24	19.90±0.55	20.47±0.65	3.78±0.12
	**2011**	95.06±3.5	22.25±0.32	20.23±0.48	19.50±0.76	3.77±0.08
	**2012**	105.46±3.8	20.55±0.27	19.74±0.63	22.47±0.41	3.80±0.13
**BF**	**2010**	116.44±4.1	21.99±0.24	20.12±0.42	21.01±0.53	3.74±0.23
	**2011**	115.65±3.3	22.25±0.32	20.26±0.58	16.97±0.75	3.81±0.10
	**2012**	118.93±5.2	20.55±0.27	20.92±0.58	22.26±0.45	3.83±0.16

Data were listed as mean ± SE

### 2. Dynamic variation of soil respiration and its components

**Fig 2:**
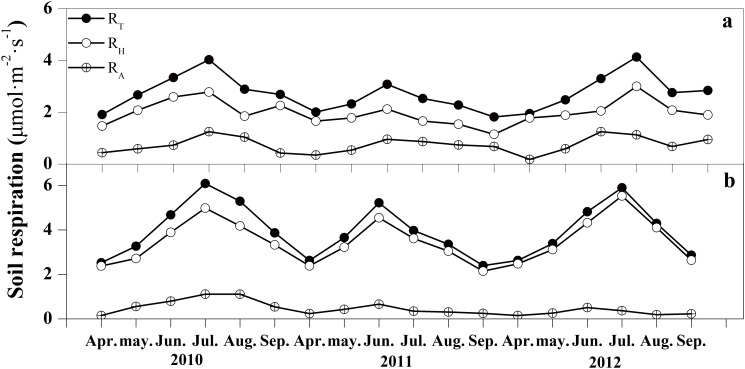
The dynamic variation of the monthly average values of total soil respiration, heterotrophic respiration and autotrophic respiration for the two forest stands.

To quantify soil respiration components, root pruning and litter removal treatments were applied to plots within both forest stands. The *R*_A_ and *R*_H_ were calculated using equations (4) and (5). Monthly-average values of soil respiration components of both forest stands varied with similar trends having single-peak curves peaking in June or July. In particular, *R*_T_ and *R*_H_ peaked in the same month in all three years in both stands, while *R*_A_, *R*_T_ and *R*_H_ peaked in the same moth during 2010 and 2011, although *R*_A_ peaked in June, before *R*_T_ and *R*_H_ peaked in 2012(Fig. 2). In addition, the soil respiration components varied obviously between the two forest stands. The *R*_T_ of the of the BNMF and BF varied from 40–53% and 54–59% during all years, respectively; meanwhile, *R*_H_ varied from 40–49% and 52–55%, and *R*_A_ varied from 63–65% and 64–86%, respectively. For both forest stands, annual means of different soil respiration components exhibited significant difference (*p*<0.05), although interannual variation was insignificant. During the study, the annual mean values for all the soil respiration components in 2011 were slightly less than the other two years (*p*>0.05) (Table 4).

**Table 4:** The average values of components of soil respiration in the two forest stands for the growing seasons (April–September) from 2010 to 2012. The different letters represent differences.

Soil respiration components	BNMF (μmol·m^–2^·s^–1^)				BF (μmol·m^–2^·s^–1^)			
	2010	2011	2012	Mean	2010	2011	2012	Mean
**Total respiration**	2.92±0.42^a^	2.34±0.43 ^a^	2.91±0.38 ^a^	2.72±0.52 ^a^	4.29±0.46 ^b^	3.54±0.29 ^b^	3.98±0.35 ^b^	3.94±0.32 ^b^
**Heterotrophic respiration**	2.17±0.36^c^	1.65±0.37 ^c^	2.12±0.27 ^c^	1.98±0.47 ^c^	3.58±0.52 ^d^	3.17±0.37 ^d^	3.70±0.48 ^d^	3.48±0.41 ^d^
**Autotrophic respiration**	0.75±0.51^e^	0.69±0.53 ^e^	0.79±0.54 ^e^	0.74±0.33 ^e^	0.71±0.42 ^e^	0.37±0.55 ^e^	0.28±0.41 ^e^	0.46±0.56 ^e^

Data were listed as mean ± SE

### 3. Contribution of each component to total soil respiration

**Fig 3:**
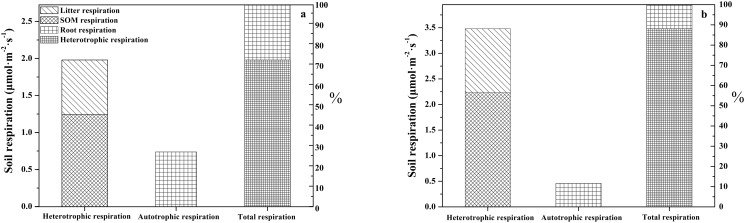
Components of soil respiration and ratios of their contributions to total soil respiration in the two forest stands.

The contributions of *R*_A_ and *R*_H_ to *R*_T_ were also determined. *R*_A_ and *R*_H_ contributed an average of 26.75% and 73.25% to *R*_T_ in BNMF, respectively, while in BF they contributed 10.98% and 89.02%, respectively (Fig. 3). As previously noted, *R*_A_ and *R*_H_ of both forests peaked in June or July, accounting for 46% and 37% of the total yearly amount of CO_2_ released from *R*_A_ and *R*_H_ in BNMF during the observation period, and 47% and 42% in BF, respectively. Equations (3) and (6) showed that decomposition of litter and organic matter accounted 36.81% and 63.19% of *R*_H_ in BNMF, and 36.43% and 63.66% in BF, respectively (Fig. 3). Overall, *R*_SOM_ made up the majority of *R*_H_ in both stands.

### 4. Relationship between components of soil respiration and temperature

The trends in the variation of soil respiration components and temperatures (AT and ST) were generally similar and positively correlated for both forest stands (Figs. 1 and 2). Statistical results show that the soil respiration components were all positively correlated to temperature. In particular, the relationship between *R*_A_ in BNMF as well as *R*_T_ and *R*_H_ in the BF with temperature was significant (*p*<0.01; Table 5). Specifically, in both forest stands the correlation coefficient (*r* value) between soil respiration components and AT was smaller than that between soil respiration components and ST. In fact, out of all the abiotic factors, ST affected soil respiration components most directly (Table 5). Meanwhile, none of soil respiration components were significantly correlated with other abiotic factors in either of the forest stands (Table 4).

**Table 5:** The correlations between abiotic environmental factors and different components of soil respiration for the two forest stands.

Forest types	Soil respirationcomponents	Environment factors									
		Air temperature		Soil temperature		Soil moisture		pH		Solar radiation	
		*r*	Sig.	*r*	Sig.	*r*	Sig.	*r*	Sig.	*r*	Sig.
**BNMF**	**Total**	0.56*	0.040	0.60**	0.008	0.26	0.291	-0.08	0.741	0.19	0.444
	**Heterotrophic**	0.47*	0.039	0.48*	0.0391	0.38	0.214	-0.36	0.887	0.14	0.579
	**Autotrophic**	0.60**	0.008	0.74**	<0.001	-0.14	0.580	-0.12	0.634	0.22	0.388
**BF**	**Total**	0.65**	0.004	0.65**	0.003	0.30	0.218	0.14	0.580	0.34	0.174
	**Heterotrophic**	0.63**	0.005	0.69**	0.001	0.33	0.188	0.15	0.542	0.36	0.136
	**Autotrophic**	0.46*	0.040	0.47*	0.043	0.14	0.587	0.05	0.859	0.13	0.608

* represents *p*< 0.05;

** represents *p*< 0.01;

*r* represents the correlation coefficient.

**Fig 4:**
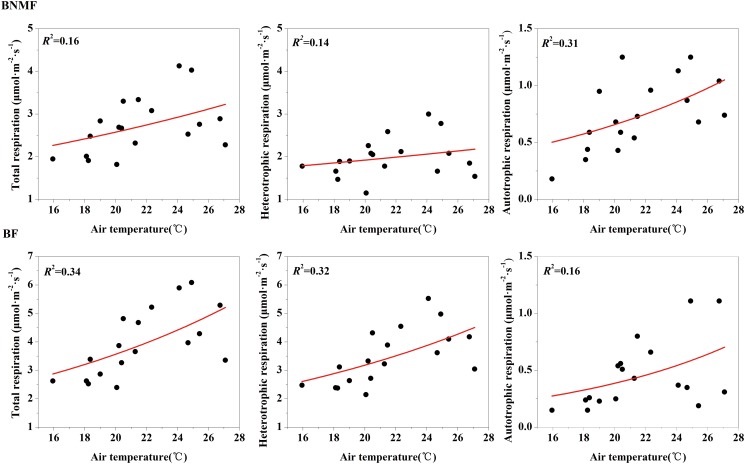
The relationship between air temperature and total soil respiration, heterotrophic respiration and autotrophic respiration, for the two forest stands.

**Fig 5:**
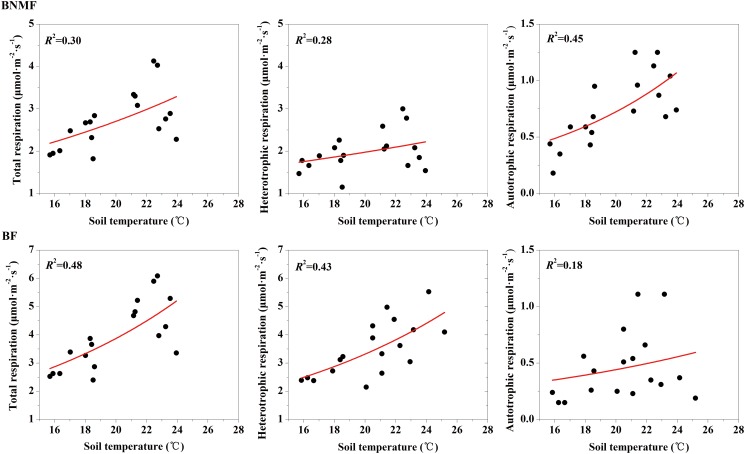
The relationship between soil temperature and total soil respiration, heterotrophic respiration and autotrophic respiration, respectively, for the two forest stands.

We used Equation (7) to describe the relationship between temperatures and soil respiration components. *R*_A_ and *R*_H_ of both forest stands increased exponentially with increasing temperature, and the explanatory ability of temperature on different soil respiration components varied (Figs. 4 and 5) The explanatory ability of AT on *R*_A_ (31%) was better than that on *R*_T_ (14%) and *R*_H_ (16%) in BNMF (Fig. 4). In contrast, AT only explained 16% of the variation of *R*_A_ for the BF stand, which was significantly less than that for *R*_T_ (34%) and *R*_H_ (32%) (Fig. 4). Similarly, explanatory ability of ST on *R*_A_ (45%) was better than that of the two other soil respiration components in BNMF stand (Fig. 5); ST explained more than 40% of the variation in both *R*_T_ and *R*_H_ (Fig. 5).

**Fig 6:**
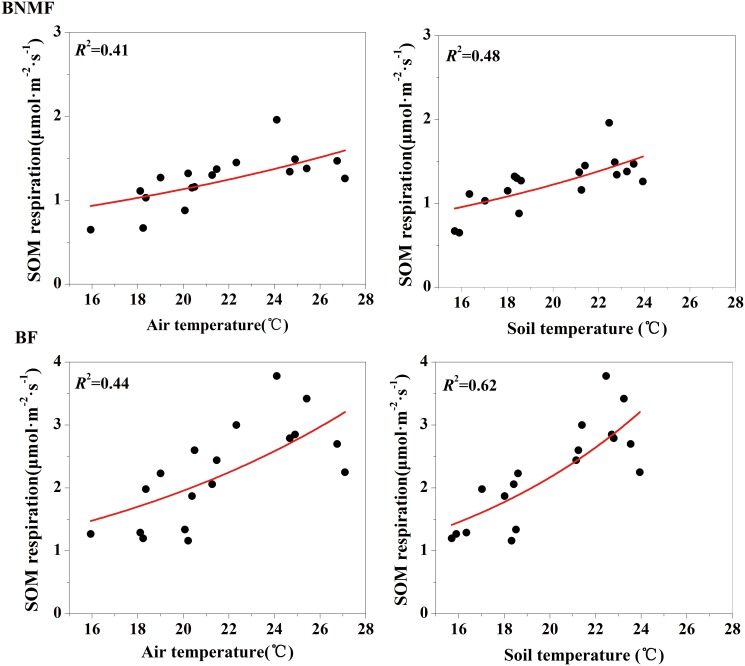
The relationship between soil organic matter and both air and soil temperatures for the two forest stands.

We also analyzed the relationship of *R*_L_ and *R*_SOM_ in both forest stands with AT and ST. The results showed that *R*_SOM_ was significantly positively correlated with AT and ST in both forests (*p*<0.01), while *R*_L_ had no significant correlation with AT or ST (Table 6). Equation (7) was used again to analyze the relationship of *R*_SOM_ with temperatures in each forest. We found that AT explained 41% and 44% of variation in *R*_SOM_ of BNMF and BF, respectively, while ST explained 48% and 62%, respectively (Fig. 6). In summary, temperature was a major factor influencing R_SOM_ in both forest stands. Since *R*_SOM_ also represented the majority of *R*_H_ in both forest stands, changes in *R*_SOM_ directly affected *R*_H_, indicating a mechanism by which temperatures generally influenced *R*_H_.

**Table 6:** The relationships between both temperature and litter respiration and soil organic matter respiration, respectively, for the two forest stands.

Forest types	Temperatures	Heterotrophic respiration component			
		Litter respiration		SOM respiration	
		*r*	Sig.	*r*	Sig.
**BNMF**	**Air temperature**	-0.15	0.553	0.73**	0.001
	**Soil temperature**	-0.28	0.254	0.69**	0.001
**BF**	**Air temperature**	0.09	0.713	0.81**	<0.000
	**Soil temperature**	0.09	0.698	0.72**	0.001

* represents p< 0.05;

** represents p< 0.01;

*r* represents the correlation coefficient.

### 5. Difference in components of soil respiration between two forest stands

**Fig 7:**
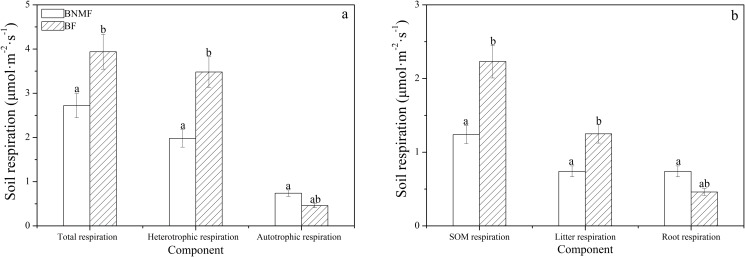
Analysis of the differences of the same component respiration of the two forests using analysis of variance (ANOVA).

Previously in this study it was proven that the degree of correlation between the identical soil respiration components and temperatures varied in different forest stands (Table 5). In addition, forest stands also affected the annual average values of soil respiration components, meaning that the ratios of soil respiration components in the two forest stands differed greatly. The average values for *R*_T_ and *R*_H_ were 3.94 ± 0.32μmol·m^–2^·s^–1^ and 3.48 ± 0.41μmol·m^–2^·s^–1^ in BF stand, respectively, and were significantly larger than those of BNMF (*p*<0.05) (Table 4, Fig. 7a). Conversely, the average values of *R*_A_ (0.74 ± 0.33μmol·m^–2^·s^–1^) in BNMF were slightly larger than that of the BF stand (0.46 ± 0.56μmol·m^–2^·s^–1^) (*p*>0.05) (Table 4, Fig. 7a). Moreover, no significant difference was observed between the *R*_L_ values of both forest stands (*p*>0.05), while the difference between *R*_SOM_ values of both forest stands was significant (*p*<0.05) (Fig. 7b).

## Discussion

### 1. Variations of soil respiration components

Butler et al. found that the monthly average components of soil respiration in central Brazil were all represented in single-peaked curves; in particular, total soil respiration and heterotrophic respiration in two forests exhibited similar trends, which peaked at the same time [14], similar to results found for both forest stands in our study (Fig. 2). However, although Kominami found that while total soil respiration and heterotrophic respiration had single-peak curves, heterotrophic respiration lagged behind that of total soil respiration in a Japanese temperate forest [35]. Litter respiration was a major component of heterotrophic respiration (88%), suggesting that heterotrophic respiration tended to peak later, because the soil C used by microorganisms during litter decomposition was not absorbed immediately. In this way, the combination of the use of C by microorganisms and the high C content of litter respiration led to the hysteresis phenomenon. This may be caused by the imprecision of the methods used. The trenching method that was used to separate *R*_T_ in our experiment was able to measure soil respiration components directly and accurately. However, this method may cause an overestimation of true soil respiration rates. Bond-Lamberty et al. cut roots and found that decomposition of dead roots in a trenched sample plot produced a relative increase of 5% in the heterotrophic respiration in *Wayward Pines*. The dead roots provide a new source of SOM for microorganisms and increased heterotrophic respiration rate [36]. Lee found that the impact of root pruning could be ignored later in the study, because dead roots turned black after 2–3 months [37]. In the current study, we did not directly observe whether the dead roots decomposed completely. Instead, we avoided the effects of dead roots on soil respiration by starting to measure soil respiration rates five months after root pruning. Nevertheless, the results indicated that *R*_H_ of both forest stands in 2010 and 2012 were higher than in 2011 (Table 4), although *R*_H_ was probably overestimated in all years. The main cause may be that the dead roots did not completely decompose in the first year, and new roots grew within the plots even in the third year. Since changes of roots activity cannot be directly observed, the effect of the decomposed and new-grown roots on *R*_H_ proved hard to identify, which may lead to a poor estimation of soil respiration. In addition, the BF stands were subjected to intensive management in this study. Some studies indicated in the extensive management in bamboo forest causes above average SOM content creating a rich C source for soil microbes. Meanwhile, the complexity of composition of litter results in a low C/N ratio that is conductive to the decomposition of organic matter by soil microbial, this accelerates the turnover SOM resulting in a larger heterotrophic respiration [38]. Therefore, intensive management may be another reason why *R*_H_ was high in the BF stand. Moreover, the SOM content in BF stand was larger than others have reported in previously research [39, 40], although the age of the BF stand was older than the ages of previously studied areas (Table 1). Previous studies have also found that soil respiration has a linear positive correlation with organic matter content, and old bamboo stands will release more CO_2_ than young stands [41, 42]; therefore, the combination of stand age and SOM content may also contribute to the high *R*_H_ in the BF stand in this study.

### 2. Contributions of the components of soil respiration to total soil respiration

Numerous studies have shown that heterotrophic and autotrophic respiration contributes to total soil respiration at different rates in different forests. Globally, the root respiration contributes between 10–60% (–90%) of total soil respiration in most forest ecosystems [43]. The present study did not distinguish root respiration from other autotrophic respiration components but instead treated the two as equivalent. When measured in this way, *R*_A_ in BNMF contributed 26.75% to *R*_T_, while that in the BF contributed 10.98%; these values are consistent with established global ranges. Different vegetation types and research methods may result in the different contributions of autotrophic respiration to total soil respiration among forest systems. The contribution of *R*_H_ to *R*_T_ was above 73% in both forests, obviously, *R*_H_ contributed more to *R*_T_ than *R*_A_. These contributions are higher than those identified by Shen for subtropical forests in the downstream region of Yangtze River [44], perhaps because the current study area is a nature reserve. The forest stands here featured minimal human interference, a thick litter layer and rich SOM, causing the relatively high contribution of *R*_H_. In addition, the overestimate of *R*_H_ in both forest stands resulted in a higher ratio of *R*_H_. Furthermore, our study was conducted during the 2010 to 2012 growing seasons, when the soil respiration components peaked, which also led to a higher ratio of *R*_H_. Subke et al. conducted a meta-analysis of the contribution of the soil respiration components based on literature from the past 30 years, and found the contribution of heterotrophic respiration to total soil respiration ranged from 27 to 86% for the forest systems [45]. In addition, Satomura found that both autotrophic and heterotrophic respiration increased over the growing season in Japanese temperate forests, accounting for more than 50% of the total CO_2_ released through *R*_T_ annually [46], a percentage that was higher than our results. During the growing season, environmental conditions favor plant growth and the amount of CO_2_ released from soil respiration increases. In summary, the contribution rates of soil respiration components to total soil respiration itself were not only affected by separation methods and stand structure, but were also affected by forest type and environmental factors.

### 3. Influence of temperature on soil respiration

This study proved AT and ST were the main causes of the variation observed in the soil respiration component [47]. For both forest stands, soil respiration components (except *R*_L_) were significantly positively correlation with AT and ST (Figs. 1a 2b; Fig. 2). These findings are consistent with the results from Zheng and Chen [25]; they investigated several regions in China, identified a significant correlation between soil respiration and temperature during growing seasons, and found that temperature was a leading factor influencing seasonal variations in soil respiration. Although a significant relationship existed between temperature and soil respiration components in both forest stands (Table 5 and 6), the degree of correlation (*r* value) between soil respiration components and temperature varied (correlation indices were different). Therefore, the explanatory ability of temperature on different soil respiration components were also varied (Figs. 4 and 5). Similarly, a study mixed forest in southeast Sweden found soil temperature explain 82% of root soil respiration but only 42% of total soil respiration [48]. This discrepancy may result from different mechanisms related to temperature affecting different soil respiration components [49, 50]. In general, temperature exerts an impact on autotrophic respiration mainly by affecting root respiration. For example, Tang et al. found that temperature affected the photosynthetic supply of C available for photosynthesis, thereby affecting C used for root respiration [51]. In addition, the previous study indicated the temperature can restrict root activity directly, and thus affect the autotrophic respiration [52]. Temperature effects heterotrophic respiration of SOM [53] because heterotrophic respiration is mainly caused by the decomposition and oxidation of organic matter, while the decomposition rate of organic matter is positively correlation with temperature [54]. Research studies have also shown that temperature affected the movement of air within soil as well as the activity and abundance of microorganisms; this in turn affected SOM respiration, thus finally affected the heterotrophic respiration [55, 56]. In this study, the temperature and *R*_SOM_ of both stands had a significant positive phase exponential relationship and a higher degree of correlation in BF. We believe that the temperatures can explain most changes of R_SOM_, and especially, ST can explain 62% of R_SOM_ variation (Fig. 7). We found BF also had high levels of SOM. Therefore, an increase in temperature accelerated the decomposition of organic matter; as a result, as more CO_2_ was released the rate of *R*_H_ also increased. Furthermore, in a temperate forest of Japan, Kominami found that decomposition rate of litter increased with increased temperature, and reported that litter respiration was higher in summer than in autumn [35]. However, the current study suggested that *R*_L_ was not correlated with temperature, primarily because of the differences in this study region and the associated vegetation type.

Other environmental factors, i.e. soil water content may also influence soil respiration components to some extent, although they were not obviously correlation with soil respiration. From July to September in 2011, the soil water content of these two forest stands was much lower than in the other two years (*p*<0.05; Fig. 1c). Meanwhile, in 2011, respiratory rate was lower than that during 2010 and 2012 despite the fact that each soil component of soil respiration in both forest stands did not change with changes in temperature (Fig. 2). This phenomenon could be attributed to the lower ST that suppresses the physiological process of root and microorganism that further reduced CO_2_ emissions [57]. Similar results were also reported for China’s Loess Plateau Region [58]. Moreover, this study analyzed effects of SR, soil pH and soil humidity on soil respiration. Although we did not find a direct relationship between these factors and soil respiration, some researchers have shown that solar radiation indirectly influences soil respiration via the direct impacts on photosynthesis, and soil pH had side effects on microbial activities [59, 60].

### 4. Influence of forest type on soil respiration

Our results verified the hypothesis that forest types lead to significant differences in soil respiration components; that is, significant differences in each component of soil respiration were found between the BNMF and BF stands (Fig. 2 and 7; *p*< 0.05). Similarly, Wang et al. found differences in each soil respiration component among six different forest types [61]. In the current study, because environmental factors were almost identical in two types of forest stands (Fig. 3), one can easily assume that forest type caused the observed differences in the ratios of soil respiration components. As a major component of soil respiration, previous studies found root respiration had a positive significant relationship with fine-root biomass [62]. In some regions, fine-root biomass of broadleaf and needle leaf mixed forest was larger than that in bamboo forest [63], which suggested that in those areas the root respiration of the former forest was higher than that of the latter; our results were consistent with this. In addition, differences in vegetation composition have been found to create differences in soil organic C content, litter input and decomposition rate of litter [64]. In one case where few differences in environmental factors were found, soil organic carbon had a positive correlation with soil respiration [65]. In this study, the organic content of soil in BF was higher than that of BNMF (Table 1) while differences in each environmental factor were not significant between the two forests. Forest type, which led to the differences in soil organic C content, was one of the primary reasons for the differences in soil respiration between the two forest stands. Furthermore, different tree species have different litter input and decomposition rates. These differences create differences in the rates and types of soil organic C input in a forest, and this in turn affects soil respiration and causes differences in soil respiration. Landsberg found that the litter input of BNMF was larger than that of BF, and litter decomposition more easily in broadleaf and needle leaf mixed forest making its *R*_L_ higher [66]. In this study, *R*_L_ of BF was also larger than that of BNMF, perhaps because as microbes decompose the organic substrate, soil organic C increases with the resulting greater litter input and a more rapid decomposition rate, so that the soil respiration rate also increased [67]. Based on the above analysis, the differences in soil respiration among forests may be caused by vegetation type, soil organic C content, litter input, and the rate at which litter decomposes.

## Conclusions

The present study quantitatively separated soil respiration and analyzed the relationship between the components of soil respiration and temperature in subtropical forests. The findings reveal that:

The environmental factors followed similar trends in both forest stands, and were not significantly different between forest stands. In both forest stands, *R*_T_, *R*_A_ and *R*_H_ were all broadly represented as a single-peaked curve over the course of the growing season (April-September), in a manner that tracked changes in temperature. In addition, each component of respiration was significantly different between the two forest stands (*p*<0.05).

The contributions of heterotrophic and autotrophic respiration were significantly different both between stands and from each other within the same stand. In particular, *R*_H_ accounted for the majority of *R*_T_ in both forest stands, making up 73.25% of total soil respiration in broadleaf and needle leaf mixed forest stand and 89.02% in bamboo forest stand. The contribution of *R*_A_ was notably lower and made up the remaining proportion of *R*_T_. Distinguishing among the components of heterotrophic respiration showed that CO_2_ released from the *R*_SOM_ was the greatest contributor to *R*_H_, accounting for over 63% of *R*_*H*_ in both forest stands.

Soil respiration and its components were significantly correlated with AT and ST in the current study, but not to any other environmental factors. In the bamboo forest stand, *R*_T_ and *R*_H_ were both significantly correlated with AT and ST (*p*<0.01), while in the broadleaf and needle leaf mixed forest stand, *R*_A_ was significantly correlated with both AT and ST (*p*<0.01). In summary, not only can temperature influence soil respiration and its components, but different forest types can also generate different soil respiration properties. This can be based on both micro climactic differences and on biological differences producing differences in the quality and quantity of both litter and soil organic matter, thus influencing the contributions of *R*_L_ and *R*_SOM_ to *R*_H_.

## Supporting Information

S1 Dataset S1 Dataset is the data of soil respiration and environmental factors data. This data contain two parts: 1. Environmental factors values, this part include the year (2010–2012) mean values of temperature, soil temperature, soil moisture, solar radiation and pH in the two type of forest stand 2. Soil respiration values, this part include the year (2010–2012) mean values of each components of soil respiration in two type of forest stand.
(XLSX)
